# Ancient rivers shaped the current genetic diversity of the wood mouse (*Apodemus speciosus*) on the islands of the Seto Inland Sea, Japan

**DOI:** 10.1186/s40851-022-00193-3

**Published:** 2022-06-21

**Authors:** Jun J. Sato, Kouki Yasuda

**Affiliations:** Laboratory of Zoology, Department of Biotechnology, Fukuyama University, Higashimura-cho, Aza, Sanzo, 985, Fukuyama, 729-0292 Japan

**Keywords:** Biogeography, Genome-wide high-throughput sequencing, Japanese archipelago, Large Japanese field mouse, LGM, Next-generation sequencing, Reduced-representation sequencing

## Abstract

The current distributions of organisms have been shaped by both current and past geographical barriers. However, it remains unclear how past geographical factors—currently cryptic on the sea floor—affected the current distributions of terrestrial animals. Here, we examined the effects of currently cryptic ancient rivers on current genetic differentiation of the large Japanese wood mouse, *Apodemus speciosus*, which inhabits islands in the Seto Inland Sea, Japan. Genome-wide polymorphisms were identified by GRAS-Di (Genotyping by Random Amplicon Sequencing, Direct) analysis of 92 *A. speciosus* individuals. Maximum-likelihood analysis was performed with 94,142 single nucleotide polymorphisms (SNPs) identified by GRAS-Di analyses. Ancient rivers were visualized by Geographic Information System (GIS) channel analysis. Maximum-likelihood analysis showed strong support for the monophyly of each population in the islands in the Seto Inland Sea; it also showed close relationships between Innoshima-Ikuchijima, Ohmishima-Hakatajima-Oshima, Ohmishima-Hakatajima, Ohsakikamijima-Ohsakishimojima, Kamikamagarijima-Shimokamagarijima, and Kurahashijima-Etajima islands. The principal component analyses of the SNPs also supported these relationships. Furthermore, individuals from islands located on the east and west sides of the main stream of the ancient river were clustered on each side with strong support. These phylogenetic relationships were completely congruent with the paleogeographic relationships inferred from ancient rivers. In conclusion, the findings demonstrated that the current distribution of genetically distinct island lineages was shaped by ancient rivers that are currently submerged beneath the Seto Inland Sea, Japan.

**Supplementary Information:**

The online version contains supplementary material available at 10.1186/s40851-022-00193-3.

## Background

Biogeography is the most fundamental research field in evolutionary biology to explore the geographical factors that shape the current distribution of organisms [1]. The distribution of terrestrial animals is often defined by the presence of contemporary factors on the surface of the land or water. However, the effects of cryptic geographical factors that existed in the past on the evolution of terrestrial animals have not been extensively explored. Past global climate change during the Pleistocene glacial/nonglacial cycles induced sea level fluctuations [2, 3]. Therefore, islands surrounded by shallow sea floor would have been considerably affected by changes in sea level [4, 5], whereby the current sea floor may have characteristics that contributed to the current biogeographic patterns of terrestrial animals. Although the effects of changes in the island-area and/or connectivity due to Late Quaternary sea-level oscillations on the current biodiversity were examined previously (e.g., plants [6]; insects [7]), those of currently submerged structures have not been assessed. An understanding of these cryptic geographical factors would provide novel insights into the lineage differentiation of organisms that cannot be understood in the framework of contemporary biogeography.

The Japanese archipelago is composed of 6852 islands with more than 0.1 km of coastline (Geospatial Information Authority of Japan); this provides the opportunity to explore the effects of cryptic geographical factors because of the arrangement of the islands, surrounded by shallow seas. Among the islands in the Japanese archipelago, the Seto Inland Sea (the largest inland sea in Japan) possesses more than 700 islands (Ministry of Environment, Japan). Because the mean depth of the inland sea is ca. 38 m (Ministry of Environment, Japan), the current Seto Inland Sea was not present during the last glacial period, when the sea level dropped below this depth; the global sea level was ca. 120–135 m lower than today at the last glacial maximum (LGM) [2, 3, 8]. Therefore, the origin of the sea is comparatively young, beginning in the earlier Holocene because of the marine transgression (i.e., the Jomon transgression; [9]). Previous geological and ostracod paleobiogeographical studies inferred that the islands in the Seto Inland Sea began formation because of sea water invasion at ca. 11 ka (kilo annum); the current form was established by ca. 8 ka [10–12]. However, the geological history of the islands of the Seto Inland Sea is poorly known because of limited geological information present at the sea floor. Two major paleo-drainages (hereafter referred to as ancient rivers; Additional File 1) were inferred from the seafloor topography, the Hoyo and Kitan Rivers; these may have flowed into the Pacific Ocean through Bungo (between Shikoku and Kyushu islands) and Kii (between Shikoku and Honshu islands) channels, respectively, affecting the formation process of the islands of the Seto Inland Sea [13, 14]. These ancient rivers are assumed to have already been present during LGM and played an initial role to form the islands in the transgression. Previous biogeographic studies of a plant (*Rhododendron ripense*; [15]), snails (*Semisulcospira* spp.; [16]), and fish (Japanese rosy bittering *Rhodeus ocellatus kurumeus*; [17]) in the main Japanese islands (Honshu, Shikoku, and Kyushu) suggested the influence of ancient rivers on the current population genetic structures of these species. To our knowledge, there have been no reports regarding the relationships between ancient rivers and the phylogeography of organisms inhabiting the islands in the Seto Inland Sea; the formation of this phylogeography was presumably directly influenced by ancient rivers. Clarifying the effects of the ancient river on the population genetic structure of organisms in the Seto Inland Sea requires additional lines of evidence.

Terrestrial organisms on islands are potential candidates to understand the geological relationships among islands. The large Japanese wood mouse, *Apodemus speciosus*, is an old terrestrial mammal in Japan, originating at around 6 Ma [18, 19] (see [20] for review of origins of mammals in Japan); it is distributed in major and adjacent small islands throughout the Japanese archipelago except the Ryukyu Islands [21]. It is also adapted to the deciduous forest ecosystems in the islands of the Seto Inland Sea [22, 23]. Therefore, analysis of the wood mice in these islands is expected to provide information concerning the geological history of the islands in the Seto Inland Sea. Sato et al. (2017) examined the genetic variations of the wood mouse populations in the islands of the Seto Inland Sea using a portion of the nucleotide sequence of the mitochondrial *Dloop* region (ca. 300 bp); they reported that the genetic diversity was reduced on smaller islands and that the *Dloop* haplotypes were not shared among islands [24]. These results suggested that the wood mouse populations are genetically homogeneous on each island because of genetic drift and differentiated from each other by island separation [24]. However, interrelationships among the *Dloop* haplotypes from these islands were unclear because of the low resolution related to the use of only a short fragment of the *Dloop* region (ca. 300 bp).

Because of recent progress in next-generation sequencing (NGS) technology, this methodology can be used to obtain data regarding large numbers of polymorphisms in the genome. Several genome-wide reduced-representation sequencing approaches have been developed, such as RAD-seq [25], MIG-seq [26], and GRAS-Di [27, 28]. The GRAS-Di method (i.e., Genotyping by Random Amplicon Sequencing, Direct) was first reported in conference proceedings by Toyota Motor Corporation [27, 28]; it is a PCR-based genotyping-by-sequencing technique that uses only 3-bp random multiplexed primers. Although it has attracted little attention, GRAS-Di is considered a useful alternative to other genotyping-by-sequencing methods, such as RAD-seq or MIG-seq [29]. It can detect thousands of SNPs in the genome, usually larger than the SNPs detected by MIG-seq, and does not require a large amount of high-quality DNA, in contrast to RAD-seq [29]. This method has only recently been used for various organisms in the ecological and evolutionary context, including vertebrates [29], invertebrates [30], and plants [31]. For example, Hosoya et al. (2019) examined the estuarine mangrove fishes around the Ryukyu Islands using the GRAS-Di method; they also resolved the population genetic structures among islands for some mangrove fish species, suggesting that this is a powerful method to assess intraspecific genetic variations [29]. The ability of this method to identify genome-wide polymorphisms could be informative for wood mice on the islands of the Seto Inland Sea.

In this study, we examined genetic variations among wood mice inhabiting the islands of the Seto Inland Sea using the GRAS-Di method; we tested the hypothesis that ancient rivers could explain the genetic relationships among the wood mice on these islands.

## Methods

### Samples examined in this study

**Fig. 1:**
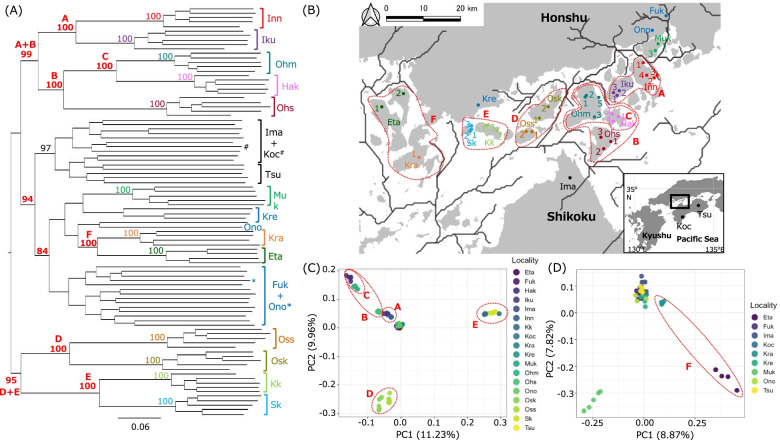
**A** Maximum likelihood tree estimated using the GTR + G model by Iqtree based on 94,142 single nucleotide polymorphisms detected in GRAS-Di analysis. Mid-point rooting was used to construct the phylogeny. The numerals above the branches are bootstrap values estimated by ultrafast bootstrap approximation (10,000 replications). These values are only shown for the clades of each island in the Seto Inland Sea and close relationships between these islands **A–F**. Full information regarding the terminal samples and bootstrap values is shown in Additional File 4. **B** Sampling localities of the examined individuals and genetic relationships (red enclosures) demarcated on the basis of the phylogenetic tree **A**. The inset in the lower right corner shows a wider map of western Japan with longitude and latitude information. Letters (**A**–**F** and each locality code) are consistent with the letters in **A**. The gray lines in the Seto Inland Sea are ancient rivers that are currently submerged, according to estimations by QGIS. The gray lines in Honshu and Shikoku are extant rivers. **C** Comparisons between PC1 and PC2 obtained by a principal component analysis with the program PLINK1.9. Letters (**A**–**E** and each locality code in the legend) are consistent with the letters in **A** and **B**. **D** The same as for **C** except for removal of individuals **A**–**E**. Letter **F** and locality code in the legend are consistent with those in **A**, **B**, and **C**

We used DNA samples from 92 individuals of the large Japanese wood mouse, *A. speciosus*, consisting of 80 samples extracted in our previous studies [24, 32] and 12 newly obtained samples from individuals in the field survey described below (Table 1). Wood mouse individuals were captured in Kure (Kre) on Honshu Island, Kurahashijima Island (Kra), and Etajima Island (Eta) using Sherman live traps baited with oats (*Avena sativa*) in 2019 (Fig. 1B; Table 1). After the mice had been captured, we collected several tissue samples using an ear punch for genetic analyses; we then released the animals to each sampling point. We obtained permission from Hiroshima Prefecture to perform the field survey and laboratory experiments on the wood mice; the study protocol was approved by the Animal Care and Use Committee of Fukuyama University (H30-Animal-8). We also followed the guidelines of the Procedure of Obtaining Mammal Specimens established by the Mammal Society of Japan.

**Table 1:** Samples and sequence reads examined in this study

Specimen code	Sampling locality	Collection data	Locality code^a^	Reads^b^	Filtered reads^c^	Sequence^e^
FACT11	Fukuyama University, East	2009.05.14	Fuk	610,605	464,378	3,192,959
FACT65	Fukuyama University, South	2011.05.18	Fuk	546,955	411,144	2,850,601
FACT68	Fukuyama University, West	2011.05.20	Fuk	523,769	394,501	2,610,847
FACT88	Fukuyama University, South	2011.07.27	Fuk	323,440	242,039	1,639,713
FACT89	Fukuyama University, South	2011.07.27	Fuk	403,307	302,261	2,204,778
FACT95	Fukuyama University, South	2011.10.04	Fuk	342,990	257,740	1,695,525
FACT96	Fukuyama University, South	2011.10.07	Fuk	173,663	131,241	950,228
FACT100	Fukuyama University, South	2011.11.01	Fuk	526,191	394,848	2,802,797
FACT120	Fukuyama University, South	2012.05.29	Fuk	460,987	349,220	2,208,359
FACT121	Fukuyama University, South	2012.05.29	Fuk	277,445	207,640	1,342,532
FACT134	Fukuyama University, South	2012.07.06	Fuk	582,717	437,018	3,029,354
FACT155	Fukuyama University, Central	2012.11.16	Fuk	408,609	304,913	2,031,635
YT2005-1-Ap1	Kurihara, Onomichi	2005.03.05	Ono	386,249	299,551	1,025,703
YT2005-1-Ap2	Kurihara, Onomichi	2005.03.05	Ono	1,145,627	882,220	4,580,390
YT2005-1-Ap4	Kurihara, Onomichi	2005.03.05	Ono	813,412	615,202	3,756,417
KKE2019-5^d^	Kawajiri, Kure	2019.03.16	Kre	184,484	138,460	936,406
KKE2019-6^d^	Kawajiri, Kure	2019.03.16	Kre	369,578	278,630	2,136,055
KKE2019-8^d^	Kawajiri, Kure	2019.03.16	Kre	417,047	313,603	2,463,980
KKE2019-11^d^	Kawajiri, Kure	2019.03.16	Kre	344,682	259,770	1,988,195
FACT110	Mukaishima 1	2012.03.19	Muk	443,391	330,374	2,213,708
FACT111	Mukaishima 1	2012.03.19	Muk	530,764	392,925	2,691,564
YT2004-Ap1	Mukaishima 3	2004.01.28	Muk	802,243	617,739	2,983,375
YT2004-Ap17	Mukaishima 3	2004.01.29	Muk	925,945	720,928	4,019,596
YT2004-Ap18	Mukaishima 3	2004.01.29	Muk	648,414	491,681	3,214,390
FACT24	Innnoshima 1	2009.08.25	Inn	601,395	447,827	3,297,068
FACT157	Innnoshima 1	2013.03.30	Inn	682,230	512,792	3,581,029
FACT158	Innnoshima 2	2013.03.30	Inn	444,213	329,269	2,423,674
YT2004-Ap2	Innnoshima 3	2004.01.28	Inn	590,038	448,767	3,062,029
YT2004-Ap9	Innnoshima 4	2004.01.28	Inn	340,931	259,071	1,751,508
FACT159	Ikuchijima 1	2013.04.23	Iku	337,184	253,724	1,838,443
FACT164	Ikuchijima 2	2013.04.30	Iku	365,148	273,741	2,018,363
FACT165	Ikuchijima 2	2013.04.30	Iku	296,847	222,520	1,689,762
FACT173	Ikuchijima 3	2013.05.07	Iku	407,486	308,867	2,325,840
FACT174	Ikuchijima 3	2013.05.07	Iku	890,895	680,129	3,855,503
FACT188	Ohmishima 1	2014.03.28	Ohm	609,670	459,241	3,148,761
FACT190	Ohmishima 2	2014.03.28	Ohm	542,077	406,988	3,022,620
FACT192	Ohmishima 3	2014.03.28	Ohm	428,164	320,154	2,521,039
FACT194	Ohmishima 3	2014.03.28	Ohm	347,202	260,088	2,111,406
YT2005-Ap43	Ohmishima 5	2005.12.16	Ohm	519,501	394,926	2,938,063
FACT203	Hakatajima 1	2014.04.19	Hak	431,393	321,996	2,471,386
FACT210	Hakatajima 3	2014.04.26	Hak	234,729	175,184	1,361,140
FACT215	Hakatajima 4	2014.05.01	Hak	362,459	273,737	2,207,141
FACT216	Hakatajima 4	2014.05.01	Hak	479,212	361,246	2,603,294
FACT217	Hakatajima 4	2014.05.01	Hak	540,473	409,670	3,039,106
FACT218	Ohshima 1	2014.05.17	Ohs	478,380	358,778	2,645,564
FACT228	Ohshima 1	2014.05.17	Ohs	351,081	262,959	2,070,297
FACT230	Ohshima 2	2014.05.17	Ohs	907,618	702,505	4,182,623
FACT231	Ohshima 2	2014.05.17	Ohs	343,885	258,180	2,060,732
YT2004-Ap65	Ohshima 3	2004.01.31	Ohs	824,801	624,690	3,319,858
YT2006-Ap12	Ohsakikamijima 1	2006.12.21	Osk	809,828	613,162	3,881,513
YT2006-Apo13	Ohsakikamijima 1	2006.12.21	Osk	638,800	493,364	3,340,137
YT2006-Apo14	Ohsakikamijima 1	2006.12.21	Osk	513,099	391,261	2,772,793
YT2006-Ap15	Ohsakikamijima 2	2006.12.21	Osk	624,672	482,994	3,389,840
YT2006-Apo16	Ohsakikamijima 2	2006.12.21	Osk	703,884	544,903	3,536,874
FACT296	Ohsakishimojima 1	2015.05.16	Oss	674,877	501,386	3,412,139
FACT298	Ohsakishimojima 1	2015.05.16	Oss	869,970	669,627	4,298,248
FACT299	Ohsakishimojima 1	2015.05.16	Oss	1,057,143	813,733	4,723,477
FACT337	Ohsakishimojima 2	2015.05.30	Oss	375,107	282,977	2,012,430
FACT338	Ohsakishimojima 2	2015.05.30	Oss	1,141,604	875,120	4,768,518
FACT301	Kamikamagarijima 1	2015.05.16	Kk	487,512	368,339	2,829,217
FACT304	Kamikamagarijima 1	2015.05.16	Kk	349,187	261,167	2,047,916
FACT347	Kamikamagarijima 2	2015.05.30	Kk	1,582,305	1,197,292	5,774,382
FACT348	Kamikamagarijima 2	2015.05.30	Kk	528,843	398,074	3,028,705
YT2006-Apo51	Kamikamagarijima 3	2006.12.23	Kk	850,356	654,643	3,181,260
FACT309	Shimokamagarijima 1	2015.05.16	Sk	562,118	424,020	3,035,563
FACT310	Shimokamagarijima 1	2015.05.16	Sk	652,773	498,554	3,406,747
FACT352	Shimokamagarijima 2	2015.05.30	Sk	528,294	398,328	3,002,296
FACT353	Shimokamagarijima 2	2015.05.30	Sk	630,449	475,138	3,241,963
YT2006-Ap91	Shimokamagarijima 3	2006.12.24	Sk	819,549	628,697	3,279,102
KKE2019-2^d^	Kurahashijima 1	2019.03.16	Kra	412,653	308,736	2,401,222
KKE2019-15^d^	Kurahashijima 1	2019.06.01	Kra	241,634	182,177	1,443,335
KKE2019-16^d^	Kurahashijima 1	2019.06.01	Kra	272,576	205,352	1,588,854
KKE2019-17^d^	Kurahashijima 1	2019.06.01	Kra	297,317	223,525	1,803,011
KKE2019-1^d^	Etajima 1	2019.03.16	Eta	480,989	359,975	2,815,332
KKE2019-25^d^	Etajima 1	2019.06.01	Eta	231,987	173,884	1,349,540
KKE2019-20^d^	Etajima 2	2019.06.01	Eta	277,509	205,728	1,658,799
KKE2019-21^d^	Etajima 2	2019.06.01	Eta	340,125	252,617	2,065,899
FACT265	Tamagawa, Imabari	2014.10.08	Ima	277,880	211,743	1,326,597
FACT266	Tamagawa, Imabari	2015.02.27	Ima	290,853	216,641	2,278,718
FACT268	Tamagawa, Imabari	2015.02.27	Ima	452,811	342,996	2,334,059
FACT269	Tamagawa, Imabari	2015.02.27	Ima	564,866	425,470	2,997,828
FACT270	Tamagawa, Imabari	2015.02.27	Ima	287,971	213,685	1,489,619
FACT271	Tamagawa, Imabari	2015.02.27	Ima	369,675	275,842	2,186,221
FACT273	Tamagawa, Imabari	2015.02.27	Ima	524,129	392,358	2,900,232
FACT276	Tamagawa, Imabari	2015.02.27	Ima	367,933	276,014	2,059,745
FACT284	Tamagawa, Imabari	2015.02.27	Ima	757,705	570,727	3,777,165
HS53	Saga, Kurose, Kochi	1984.11.12	Koc	288,082	215,310	1,512,866
HS310	Mt. Tsurugi, Tokushima	unknown	Tsu	545,818	409,244	2,996,747
HS2222	Mt. Tsurugi, Tokushima	unknown	Tsu	468,929	348,670	2,616,918
HS2223	Mt. Tsurugi, Tokushima	unknown	Tsu	938,752	700,751	4,372,891
HS2797	Mt. Tsurugi, Tokushima	unknown	Tsu	809,282	609,439	3,825,308
HS2798	Mt. Tsurugi, Tokushima	unknown	Tsu	430,746	323,737	2,447,166

^a^The locality codes are consistent with those in Fig. 1

^b^Total reads obtained from NGS

^c^Filtered reads with more than Q30 quality and with length more than100 bp

^d^Samples newly obtained in this study

^e^Sequences examined in STACKS for each sample (base pair)

### DNA extraction, PCR, and sequencing

DNA was extracted from ear tissue samples using a DNeasy Blood & Tissue Kit (Qiagen, Hilden, Germany), in accordance with the manufacturer’s instructions. The extracted DNA concentration was calculated using a NanoDrop spectrophotometer (Thermo Fisher Scientific, Waltham, MA, USA) and used to determine the amount of DNA input for PCR analyses.

Two-step PCR was used to construct a library for NGS via the GRAS-Di method. Two consecutive rounds of PCR were performed using a PrimeSTAR® HS DNA Polymerase kit (Takara, Kusatsu, Japan) on a LifeECO thermal cycler (Bioer Technology, Hangzhou, China). The first PCR mixture (25 μL) contained 5 × PrimeSTAR Buffer (Mg^2+^), 0.2 mM of each dNTP, 40 pM of multiplexed primer mix (for each random primer, see Additional File 2), PrimeSTAR HS DNA Polymerase (0.625 U/sample), template DNA (15 ng), and PCR-grade water. The first round of PCR was performed with an initial denaturation step at 98 °C for 2 min, followed by 30 cycles of denaturation at 98 °C for 10 s, annealing at 50 °C for 15 s, and extension at 72 °C for 20 s.

The second round of PCR was performed directly on PCR products obtained from the first round of PCR in a mixture (50 μL) containing 5 × PrimeSTAR Buffer (Mg^2+^), 0.2 mM of each dNTP, 0.25 pM of forward (AATGATACGGCGACCACCGAGATCTACAC-Index2-TCGTCGGCAGCGTCAGATGTGTATAAGAGACAG) and reverse (CAAGCAGAAGACGGCATACGAGAT-Index1-GTCTCGTGGGCTCGGAGATGTGTATAAGAGACAG) primers (where Index 1 and 2 are 8-bp strings of bases for demultiplexing samples after the NGS run), PrimeSTAR HS DNA Polymerase (1.25 U/sample), product from the first round of PCR (1.5 μL), and PCR-grade water. The 3′-end sequences of each primer were designed to prime the primer regions from the first round of PCR. The 5′-end sequences of each primer were adapters for binding to the Illumina NGS flow cell (Illumina, San Diego, CA, USA). The second round of PCR consisted of an initial denaturation step at 95 °C for 2 min, followed by 25 cycles of denaturation at 98 °C for 15 s, annealing at 55 °C for 15 s, and extension at 72 °C for 20 s, with a final extension at 72 °C for 1 min. Products from the second round of PCR were mixed in equal volumes and purified using a Mini Elute PCR Purification Kit (Qiagen).

The concentrations of the libraries were assessed using a Synergy H1 microplate reader (BioTek, Winooski, VT, USA) with QuantiFluor dsDNA System (Promega, Madison, WI, USA); the qualities of these libraries were examined using a Fragment Analyzer (Agilent Technologies, Santa Clara, CA, USA) with a dsDNA 915 Reagent Kit (Agilent Technologies). Finally, NGS was performed using an Illumina HiSeq X Ten with HiSeq X Ten Reagent Kit v2 (150 cycles, paired-end). Raw FASTQ data for each sample generated from the NGS run were deposited in the DDBJ SRA database (DRA013162). Experiments from PCR to sequencing were performed by Bioengineering Lab. Co., Ltd. (Sagamihara, Japan).

### Data filtering and SNP calling

For FASTQ data generated from the NGS run and demultiplexed into each individual, Sickle version 1.33 [33] was used to trim sequence data into only data with quality > Q30 and length > 100 bp. FASTX-Toolkit version 0.0.14 (http://hannonlab.cshl.edu/fastx_toolkit/index.html, Accessed 2 December 2021) was then used to unify the length of the examined sequence to 100 bp (fastx_trimmer).We then used Bowtie2 version 2.3.4.1 [34] to map these trimmed sequences to the reference genome of *Apodemus speciosus* (Accession code in NCBI: Aspe_assembly01 [35]) and obtained “.sam” file, followed by conversion of “.sam” into sorted “.bam” file in Samtools version 1.7 [36]. With these sorted “.bam” files, we applied STACKS version 2.59 [37, 38] with the commands “ref_map.pl” and “populations” to obtain the SNPs among samples and the input files for the subsequent phylogenetic and principal component analyses. We extracted a randomly selected SNP from each locus using the “write-random-snp” option in “populations”. We also omitted SNPs sites that showed more than 0.5 heterozygosity and less than 0.05 mean allele frequency. Other parameters in the STACKS were set by default.

### Phylogenetic inferences

For phylogenetic analysis, Iqtree version 2.0.3 [39] was used to construct the maximum likelihood tree with the GTR + G substitution model; nodal support in the phylogenetic tree was evaluated using the Ultrafast bootstrap proportion method (10,000 replications) [40]. Because there was no outgroup information in our samples, the midpoint rooting method was used to construct the phylogeny.

### Principal component analysis

Using the extracted SNPs data from STACKS, we performed a principal component analysis (PCA). We obtained “.map” and “.ped” files generated by STACKS for examining the PCA in the program PLINK1.9 (www.cog-genomics.org/plink/1.9/, Accessed 10 January 2022; [41]). PLINK produces eigen vector and eigen value files. We then used *tidyverse* library in the *R* environment [42] to depict the PCA graphs with *ggplot* function.

### Assessment of correlation between genetic and geographic distances

To assess the correlation between genetic and geographic distances, we compared *p*-distances calculated in MEGA X [43] based on the SNP data and geographic distances calculated from coordinates (Additional File 3) captured in QGIS version 3.4.12 [44]. The calculation of the geographic distances from coordinates was performed with *geosphere* library in the *R* environment. We used *vegan* library in *R* to conduct a Mantel test to evaluate the statistical significance between genetic and geographic distances with the *pearson* method and 9999 permutations.

### Visualization of ancient rivers

To visualize ancient rivers from the geological data, channel analysis was performed using QGIS and SAGA GIS version 2.3.2 [45]. The dataset used in this analysis was GEBCO_2019 Grid [46], a continuous terrain model for ocean and land with a spatial resolution of 15 arc seconds. This dataset is composed of digital elevation data obtained by satellites, bathymetry data obtained with multi-beam sonar from ships, or the sea floor topography estimated from satellite altimeters. The SAGA GIS hydrological modeling tool was used for channel analysis. The tool Fill sinks xxl [47] was used to fill surface depressions in the Digital Elevation Model, thereby generating a depression-less elevation layer. A Strahler stream order raster was created from the filled elevation data using the Strahler order tool. Finally, using the SAGA GIS tool, Terrain Analysis Channels, tributaries with Strahler’s stream order 4–8 were extracted.

## Results

### Extracted data

From GRAS-Di sequencing with the Illumina NGS platform, we obtained a mean (standard deviation) of 531,306 (242,846) total reads among the 92 samples examined (Table 1). These data were filtered for quality > Q30 and length > 100 bp. Consequently, the filtered data yielded a mean (standard deviation) of 401,983 (186,770) reads (Table 1). Sequences examined in the STACKS analyses had a mean (standard deviation) length of 2,688,332 (932,656) bp (Table 1).

### Phylogenetic relationships

Through SNP-calling in STACKS analyses, we obtained 94,142 SNPs among 92 individuals. The maximum likelihood tree inferred from these 94,142 SNPs showed that all individuals on each island in the Seto Inland Sea were clustered (Fig. 1A). These clades were supported with high bootstrap values (100%; Fig. 1A; Additional File 4). Individuals in the larger Shikoku Island (Ima, Koc, and Tsu) were also clustered in a clade with high bootstrap value (97%; Fig. 1A; Additional File 4).

With regard to the interrelationships among island individuals, we observed six highly supported relationships among adjacent islands (Fig. 1A and B; labeled A–F): A, Inn (Innoshima)-Iku (Ikuchijima); B, Ohm (Ohmishima)-Hak (Hakatajima)-Ohs (Ohshima); C, Ohm-Hak; D, Osk (Ohsakikamijima)-Oss (Ohsakishimojima); E, Kk (Kamikamagarijima)-Sk (Shimokamagarijima); F, Kra (Kurahashijima)-Eta (Etajima). These relationships were supported by high bootstrap values (100%; Fig. 1A; Additional File 4). Additionally, individuals from eastern islands (A + B; 99% for Inn, Iku, Ohm, Hak, and Ohs) and western islands (D + E; 95% for Osk, Oss, Kk, and Sk) located in each side of the main stream of the hypothetical ancient Hoyo river were clustered in each clade with strong support (Fig. 1A; Additional File 4). Individuals from Muk (Mukaishima) and Kra-Eta were not included in the eastern and western clades, respectively, and were closely related to those from Honshu (Fuk, Ono, and Kre). See Additional File 4 for all bootstrap values in the phylogeny.

### Principal component analysis

The result of the PCA analysis was consistent with that observed in the phylogenetic analyses (Fig. 1C). Five of six clusters that were observed in the phylogeny (A, B, C, D, E in Fig. 1A) were also clustered in the PCA plot (Fig. 1C). The cluster F including Kra and Eta individuals was weakly supported in the comparison excluding the individuals within the clade A, B, C, D, and E (Fig. 1D).

### Correlation between genetic and geographic distances

There was no correlation between genetic and geographic distances (Additional File 5). Mantel test provided a coefficient of *r* = -0.08681, which was statistically not significant (*P* = 0.9413). These results show that the close relationships among island lineages cannot be explained by geographical proximity between the islands in the Seto Inland Sea.

### Ancient rivers detected

Through channel analyses, we detected ancient rivers that were presumed to be present in the Seto Inland Sea. We assessed the Strahler’s stream order through 4–8 (Additional File 6) and focused on major streams by combining streams concerned with the island relationships for visual reasons (Fig. 1B). See Additional File 6 for precise information of extracted rivers from each Strahler’s stream order. The major stream (hypothesized to be an ancient river known as Hoyo River) was found to have divided several islands; their division patterns were completely congruent with the phylogenetic relationships estimated above (Fig. 1A and B).

## Discussion

Compared with our previous study using a portion of the mitochondrial *Dloop* sequence (ca. 300 bp; [24]), we obtained much a larger data set (2,688,332 bp) for phylogenetic analysis. The number of SNPs (94,142) extracted from these data was much greater than that in a previous study of estuarine mangrove fishes, which detected 4000–9000 SNPs after quality filtering [29]. This may be because we used reference-genome based SNP calling. Our initial analysis with de novo SNP calling provided 7678 SNPs that were consistent with those in the previous study. The large number of SNPs generated by the GRAS-Di analysis provided novel findings concerning the implications of the ancient river in shaping the current genetic diversity of *A. speciosus* on the islands of the Seto Inland Sea.

We used a concatenated SNPs dataset for the phylogenetic analyses. Concatenating the data might ignore coalescent variance and assume that all loci share the same genealogy [48]. Especially, incomplete lineage sorting (ILS) is often a problem for inferring the phylogenetic relationships. However, the phylogenetic node where the ILS is concerned is an “anomaly zone”, which has a tight span between consecutive divergences (e.g., due to rapid radiation) and in a condition in which the ancestral population size was large [49]. Such a node is difficult to resolve using either concatenation or ILS-aware methods, often producing low support values. As inferred from the high bootstrap values obtained in this study, the supported relationships should not have been affected by the ILS. Furthermore, considering the small population sizes in the island populations as inferred from the low genetic diversity of each island population compared with the Honshu or Shikoku populations [24], it is not likely that ancestral polymorphisms have been considerably maintained in such a small population as observed in mtDNA analyses where haplotypes in islands were not observed in Honshu and Shikoku [24]. Moreover, it is difficult to detect the ILS in this study because a small fragment sequence of less than 150 bp from each locus (HiSeq sequencer produces 150 bp at maximum) would have poor phylogenetic information [50]. In other words, the topology obtained from the concatenated sequences could not be rejected by any gene-tree discordances from each locus. It should also be noted that there is much hidden phylogenetic information in each locus that would not be emergent in the single locus analysis [50]. Using the concatenation method that would reveal the hidden information in each locus, a correct phylogenetic relationship would be supported even under the presence of gene tree discordance [50]. Extraction of only one SNP randomly from each locus might provide open and/or hidden phylogenetic information, enabling fair analyses. For these reasons, we performed a concatenated phylogenetic analysis.

As shown in Fig. 1B, we found complete consistency between phylogenetic affinity among some island lineages and the geographic relationship predicted by ancient rivers that were detected by channel analyses. The PCA analyses also provided consistent results (Fig. 1C, D). Overall, the results strongly support the hypothesis of the presence of the ancient Hoyo River flowing on the west side of the Seto Inland Sea. Although it is not clear that the fluid water flowed in the cold environment during LGM at around 20 ka, the channel analysis using sea level 120 m lower than today suggested the presence of the structure of ancient rivers in the Seto Inland Sea at that time (Additional file 1). We therefore assumed that the gene flows should have been restricted by the ancient rivers during the LGM. To supplement the geological evidence, which has been difficult to explore without large-scale investigation on the sea floor surface, the presence of ancient rivers in the Seto Inland Sea has been suggested by biological (particularly biogeographical) data. Kondo et al. (2009) examined microsatellite variations in a plant species, *Rhododendron ripense*, which has a seed dispersal strategy involving water; they suggested that populations were genetically different among river systems on Honshu, Shikoku, and Kyushu Islands that could be interconnected via two major ancient rivers in the Seto Inland Sea [15]. The current genetic structure of East Asian freshwater snails in Japan was presumably affected by past mtDNA introgressions that were facilitated by ancient rivers in the Seto Inland Sea [16]. Furthermore, Takahashi et al. (2020) showed genetic differentiation of the Japanese rosy bitterling, *Rhodeus ocellatus kurumeus*, between Honshu-Shikoku and Kyushu lineages using the Double Digest RAD-seq technique; they suggested the effect of ancient rivers on the lineage differentiation [17]. These previous studies, considered with the present study, supported the presence of the ancient river, thereby suggesting that the ancient river hypothesis is correct. Although interrelationships among the island lineages were not resolved in our previous study through the analysis of short *Dloop* sequences [24], much more abundant sequence data from the GRAS-Di analyses in the present study greatly increased the resolution concerning phylogenetic relationships among island lineages in the Seto Inland Sea. Notably, the individuals from islands on the east and west sides of the Hoyo River were closely related on each side, further supporting the ancient river hypothesis. It is also remarkable that Muk and Kra-Eta individuals were not clustered with the eastern and western clades, respectively, and instead were closely related to the Honshu individuals, also highlighting the effect of the ancient river. In future studies, application of the same methodology used in the present study for the other island populations of wood mice would illuminate the overall formation process of islands in the Seto Inland Sea. Furthermore, understanding the effects of current rivers on the genetic diversity of the wood mice would also be expected.

The observation that the island lineages were clustered in each clade in the phylogeny suggested that the effects of genetic drift through the bottleneck were stronger on the smaller islands in the Seto Inland Sea, making island-specific lineages in combination with independent mutations that occurred on each island after separation of the islands or with differential fixation of ancestral genetic variations. Such an island trend is also congruent with the results reported by Sato et al. [24], whereby the *Dloop* haplotypes detected on each island were not shared among different islands. However, the trend that the island haplotypes were more closely related to Honshu haplotypes than to Shikoku haplotypes, as observed in the *Dloop* analyses [24], was not confirmed in the present study, except for Muk and Kra-Eta islands, which would not have been separated from Honshu by ancient rivers. This may have been related to the stochastic effect caused by the low resolution of the *Dloop* sequences or the difference between mitochondrial DNA genealogy and phylogeny based on genome-wide SNP data. Further studies with more samples from Honshu and Shikoku Islands and high-throughput genome sequencing are needed to test these trends. Such studies should also clarify the effect of ancient rivers on the Honshu and Shikoku Islands. It should be further noted that effective population size of the ancestral population has affected the degree of maintenance of ancestral genetic variation, thereby explaining the long terminal branches in the phylogeny. However, it is difficult to estimate the population size parameter in this study with only a few individuals from each island. Analyses of more samples from islands would clarify the reasons for the island specificity in more detail.

## Conclusions

Using the GRAS-Di high-throughput sequencing method, we clarified the genetic diversity of the large Japanese wood mouse that would have been shaped by an ancient river, the Hoyo River. To our knowledge, this is the first study to clarify the implications of an ancient river—currently submerged on the sea floor—in the genetic differentiation of island organisms. Application of the same method to various terrestrial organisms would elucidate the geological history of the islands of the Seto Inland Sea and the mechanisms that have shaped ecosystems on each island.

## Supplementary Information

**Additional file 1.** Hypothetical ancient Hoyo and Kitan rivers. Dotted lines around the main Japanese islands show the past coastlines when the sea level dropped by 120 m.

**Additional file 2.** Random primers used in the first round of PCR.

**Additional file 3.** Geographic coordinates for samples examined in this study.

**Additional file 4.** Maximum likelihood tree estimated using the GTR+G model by Iqtree based on 94,142 single nucleotide polymorphisms detected in GRAS-Di analysis. Mid-point rooting was used to construct the phylogeny. The nodal values are bootstrap values estimated by ultrafast bootstrap approximation (10,000 replications).

**Additional file 5.** A graph showing comparison between genetic and geographic distances made by ggplot analysis in the* R* environment. See text for calculation of the genetic and geographic distances.

**Additional file 6.** Ancient rivers estimated through channel analyses using QGIS (Strahler’s stream order 4–8). Orders 4–8 (Upper left) show major streams combining rivers detected in the figures for orders 4–8.

## Data Availability

Obtained DNA sequences are deposited to DDBJ sequence read archive (SRA) in the FASTQ format under the accession number DRA013162.
