# Supplementary figures and images for: Ancient rivers shaped the current genetic diversity of the wood mouse (Apodemus speciosus) on the islands of the Seto Inland Sea, Japan
Source: Zoological Lett. 2022 Jun 21;8:9. doi: 10.1186/s40851-022-00193-3 (PMC9210816; doi:10.1186/s40851-022-00193-3)

## Slide 1
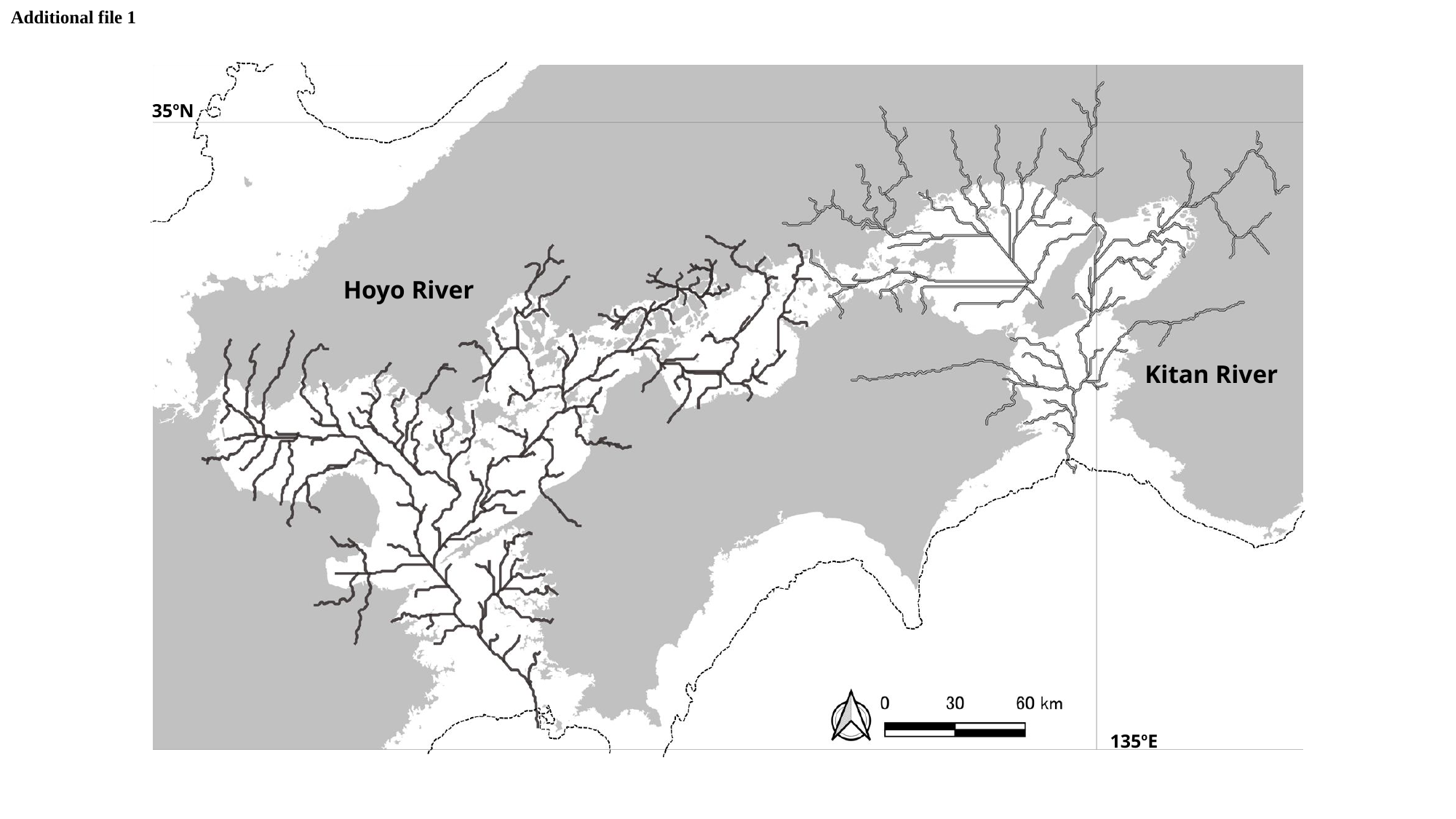

Additional file 1
35oN
Hoyo River
Kitan River
135oE

Supplement: Supplementary file 1 — Additional file 1. Hypothetical ancient Hoyo and Kitan rivers. Dotted lines around the main Japanese islands show the past coastlines when the sea level dropped by 120 m. [file 40851_2022_193_MOESM1_ESM.pptx]

## Slide 1
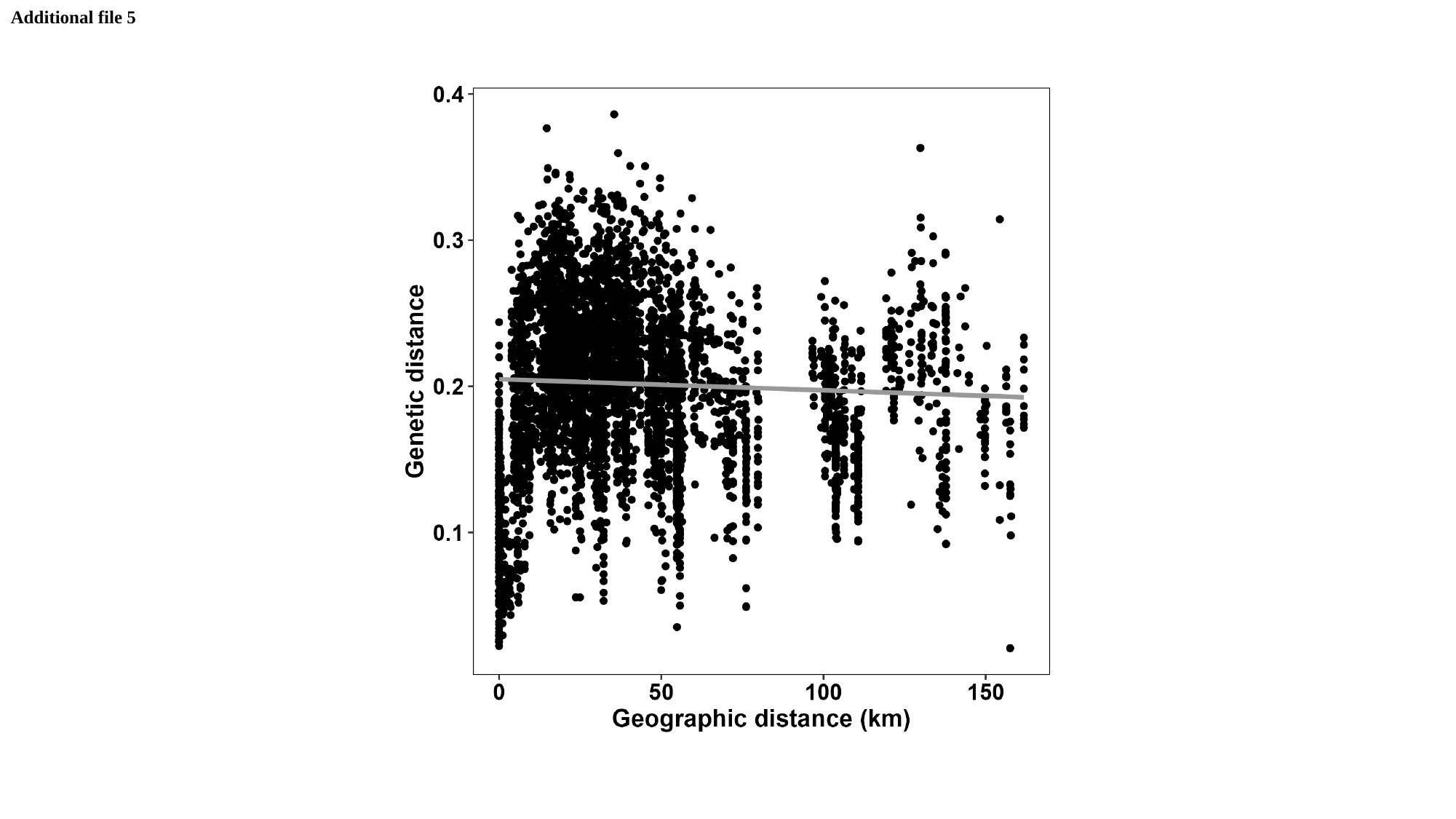

Additional file 5

Supplement: Supplementary file 5 — Additional file 5. A graph showing comparison between genetic and geographic distances made by ggplot analysis in the R environment. See text for calculation of the genetic and geographic distances. [file 40851_2022_193_MOESM5_ESM.pptx]

## Slide 1
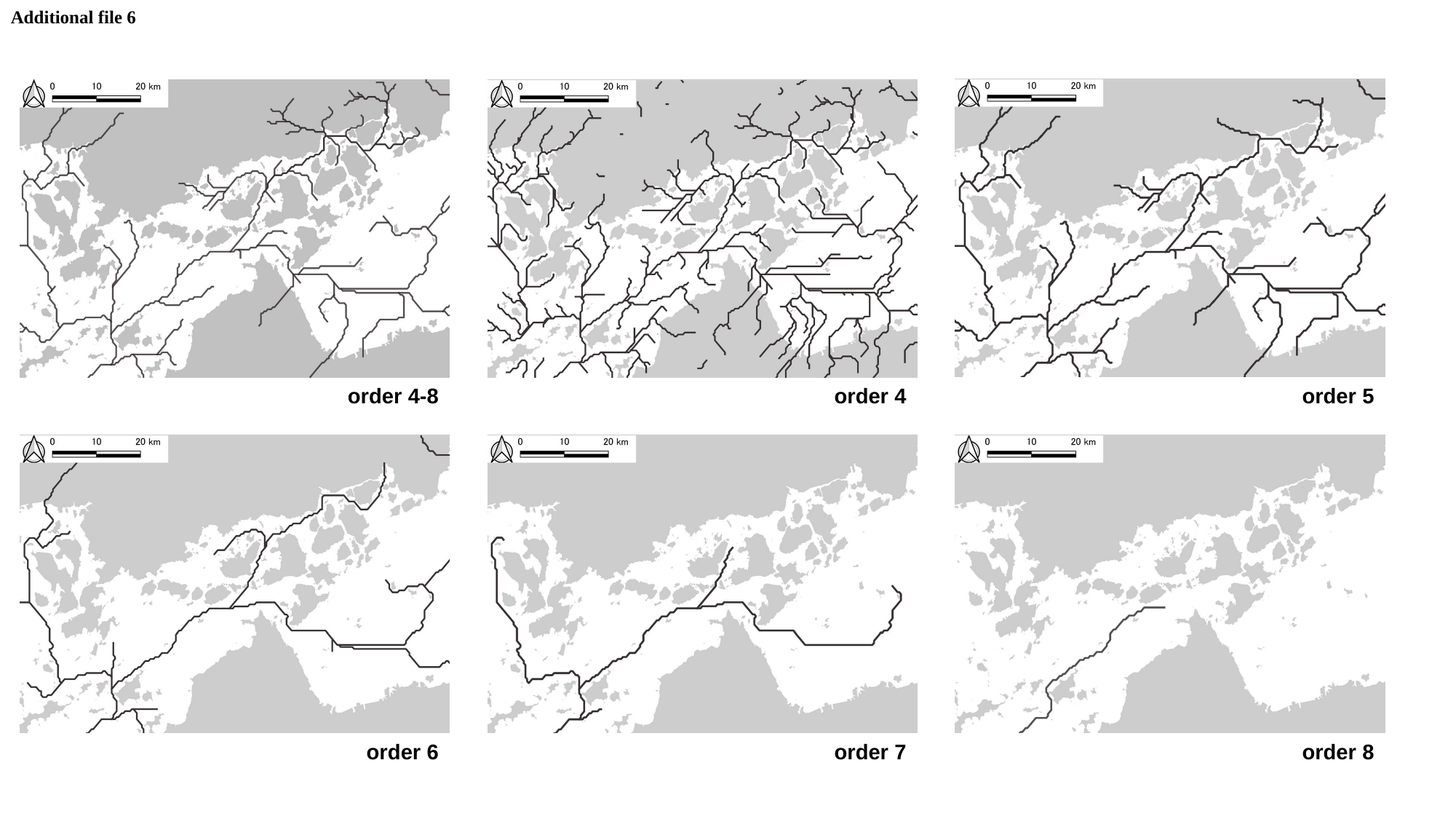

Additional file 6
order 5
order 4-8
order 4
order 8
order 6
order 7

Supplement: Supplementary file 6 — Additional file 6. Ancient rivers estimated through channel analyses using QGIS (Strahler’s stream order 4–8). Orders 4–8 (Upper left) show major streams combining rivers detected in the figures for orders 4–8. [file 40851_2022_193_MOESM6_ESM.pptx]
