# Supplementary material for: Ancient rivers shaped the current genetic diversity of the wood mouse (Apodemus speciosus) on the islands of the Seto Inland Sea, Japan
Source: Zoological Lett. 2022 Jun 21;8:9. doi: 10.1186/s40851-022-00193-3 (PMC9210816; doi:10.1186/s40851-022-00193-3)
Supplement: Supplementary file 4 — Additional file 4. Maximum likelihood tree estimated using the GTR+G model by Iqtree based on 94,142 single nucleotide polymorphisms detected in GRAS-Di analysis. Mid-point rooting was used to construct the phylogeny. The nodal values are bootstrap values estimated by ultrafast bootstrap approximation (10,000 replications). [file 40851_2022_193_MOESM4_ESM.pptx]

## Slide 1
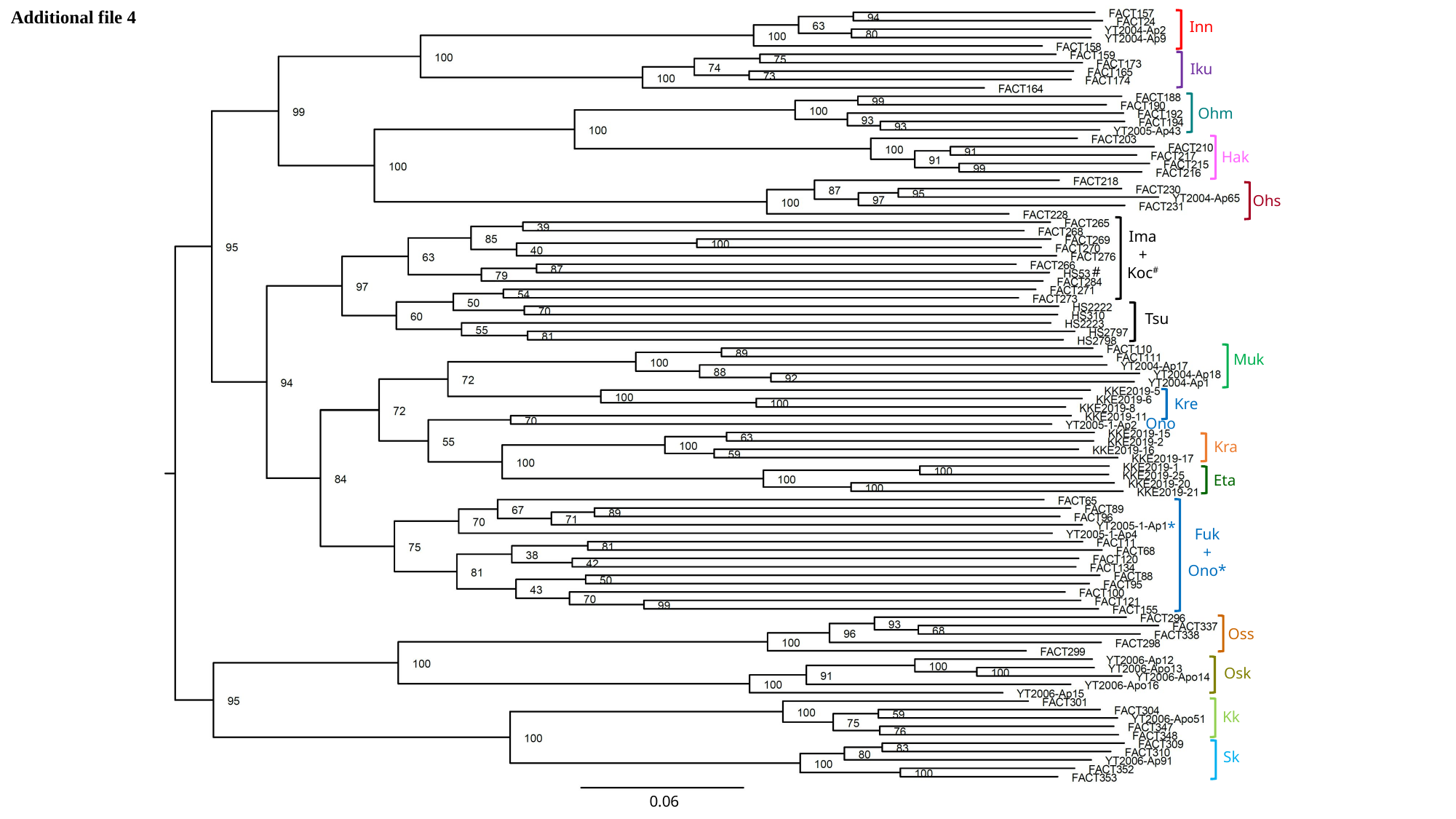

Additional file 4
Inn
Iku
Ohm
Hak
Ohs
Ima
+
Koc#
#
Tsu
Muk
Kre
Osk
Ono
Kra
Eta
*
Fuk
+
Ono*
Kk
Sk
Oss
Osk
Kk
Sk
0.06
